# Studies on Log *P_o/w_* of Quinoxaline di-*N*-Oxides: A Comparison of RP-HPLC Experimental and Predictive Approaches

**DOI:** 10.3390/molecules16097893

**Published:** 2011-09-13

**Authors:** Elsa Moreno, Elisabetta Gabano, Enrique Torres, James A. Platts, Mauro Ravera, Ignacio Aldana, Antonio Monge, Silvia Pérez-Silanes

**Affiliations:** 1 Neglected Diseases Section, Drug R&D Unit, Center for Applied Pharmacobiology Research, University of Navarra, C/ Irunlarrea s/n, 31008 Pamplona, Spain; 2 Dipartimento di Scienze dell’Ambiente e della Vita, Università del Piemonte Orientale “A. Avogadro”, Viale Michel 11, 15121 Alessandria, Italy; 3 School of Chemistry, Cardiff University, Park Place, Cardiff CF10 3AT, UK

**Keywords:** HPLC, lipophilicity, log *P*, quinoxalines

## Abstract

As reported in our previous papers, a series of quinoxaline-2-carboxamide 1,4-di-*N*-oxide derivatives were synthesized and studied as anti-tuberculosis agents. Here, the capability of the shake-flask method was studied and the retention time (expressed as log *K*) of 20 compounds were determined by RP-HPLC analysis. We found that the prediction of log *P* by the RP-HPLC analysis can result in a high accuracy and can replace the shake-flask method avoiding the experimental problems presented by quinoxaline di-*N*-oxides. The studied compounds were subjected to the ALOGPS module with the aim of comparing experimental log *P*_o/w_ values and predicted data. Moreover, a preliminary *in silico* screening of the QSAR relationship was made confirming the influence of reduction peak potential, lipophilicity, *H*-bond donor capacity and molecular dimension descriptors on anti-tuberculosis activity.

## 1. Introduction

Quinoxaline derivatives are a class of compounds that show very interesting biological properties and, therefore, they are receiving increasing attention from many medicinal chemistry researchers. The quinoxaline ring has been described as a bioisoster of quinolein, naphthalene and some other heterocycles which are the base of many antimalarial, antibacterial or antitumor agents (such as quinine, mefloquine, isoniazid, pyrazinamide or tirapazamine) [1].

The oxidation of both nitrogens of this heterocyclic system, carried out in order to obtain quinoxaline 1,4-di-*N*-oxide (QdO) derivatives, increases the number of biological properties [2]. It has been reported that QdO derivatives actually improve the biological results shown by their reduced analogues and are endowed with antiviral, anticancer [3,4], anticandidal [5,6], antibacterial [7,8,9,10,11], and antiprotozoal activities [12,13]. In fact, since the 1940s, QdOs were known as potent antibacterial agents and subtherapeutic levels have been used as animal growth promoters in feed additives [11,14,15].

Taking into account the fact that QdOs are receiving growing attention in the field of medicinal chemistry, it would be interesting to study the physicochemical properties of this family of compounds. The study of absorption, distribution, metabolism and excretion should be considered in the first stages of compounds development as this information could be of great help when identifying new candidates or optimizing structures [16,17,18,19]. The main properties for studying ADME in biological systems are solubility, lipophilicity, stability, and acid-base character. Lipid solubility is one of the most important determinants of the pharmacokinetic characteristics of a drug and many properties, such as absorption, penetration or elimination are related to lipophilicity.

The logarithm of *n*-octanol/water partition coefficient (log *P_o/w_*) is the most frequently used parameter for measuring as it has been shown that this partition system is a good model for many biological processes [16,17,20,21,22]. In fact, log *P_o/w_* is also used as one of the standard properties identified by Lipinski in the “rule of 5” for drug-like molecules [23,24].

Measurement of this parameter is always recommended and many methods have been developed for this purpose. The classical shake-flask method is time-consuming. Its use is limited in the log *P_o/w_* range between -2 and 4, and it is impossible to use with surface-active materials [25]. For these reasons, many chromatographic methods have successfully been used to assess lipophilicity of organic compounds. HPLC provides an easy, reliable and accurate way to determine the partition properties of compounds based on their chromatographic retention times [16,17,26,27]. On the other hand, since the 1970s, several methods have been proposed for log *P* computation. These methods could be divided into two groups: property-based methods and additive methods. The former group computes log *P* as a function of molecular properties such as molecular surfaces, volumes, partial charges or HOMO/LUMO energies, including topological indices as descriptors [28,29]. Additive methods firstly introduced by Hansch and co-workers [30,31,32], use basic structural building blocks as descriptors. They calculate the value of a molecule by summing up the contributions from all the blocks of the structure and considering some correction factors [26,33,34,35].

In this paper the viability of the RP-HPLC and the shake-flask methods to measure the partition coefficients of QdO is studied. Moreover, the capability of different predictive methods to properly parametrize the *N*-oxide function is evaluated.

## 2. Results and Discussion

### 2.1. Experimental log *P_o/w_*: Shake-Flask vs. RP-HPLC Method

The lipophilicity of a drug is related to its ability to cross cell membranes by means of passive diffusion. This property is usually expressed by the logarithm of the *n*-octanol/water partition coefficient, log *P_o/w_*. The log *P_o/w_* reflects the relative solubility of the drug in *n*-octanol (a model of the lipid bilayer of a cell membrane) and water (the fluid inside and outside cells). Traditionally, log *P_o/w_* values are measured using the “shake-flask” with the *n*-octanol and water partition system.

The photochemical instability of QdO is well-known and many studies have been reported [36,37,38]. It has been observed that the absorption spectrum of neutral QdO solutions change quickly due to exposure to sunlight. For these reasons, solutions of QdO must be kept protected from light and used as soon as prepared. With the aim of studying the suitability of the shake-flask method, four compounds (**10**, **14**, **22** and **26**) were selected and many attempts to measure the log *P_o/w_* were performed using the classical shake-flask method. Thus, it was verified that the classical “shake-flask” method is not suitable for the measurement of log *P_o/w_* values of QdO derivatives. In fact, these compounds have very low solubility in water and, above all, some of them degrade in solution (see supporting information for details) and create emulsions during the partition procedure.

For these reasons, a RP-HPLC method was used for the determination of log *P_o/w_* values of QdO derivatives. The RP-HPLC method used (see Experimental section) is applicable to the QdO derivatives because it is performed at pH = 7.4 and quinoxalines di-*N*-oxide derivatives are more stable in neutral solutions than working at extreme pH values. Moreover, the period of time in which the compounds are in contact with the mobile phase is not long enough for the quinoxalines to degrade as was observed when studying the corresponding chromatograms (see supporting information for details).

#### 2.1.1. Correlation between log *P_o/w_* and log *k’_0_*

In this study nine compounds (**1–9**) have been selected as reference compounds from the Recommended Reference Compounds list published by the OECD [39]. The chosen compounds allow building a model covering a fairly wide range of log *P_o/w_* values (*i.e.*, from *ca.* 0 to 4.5). The retention times of these reference compounds have been measured and expressed as log *k’_0_*. The log *k’* values have been extrapolated to 0% methanol in order to determine the capacity factors represented as log *k’_0_*. To predict the log *P_o/w_* values using the log *k’_0_* values, the least square regression was employed to generate Equation (1): 


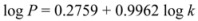
(1)


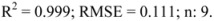


The cross-validation statistical parameters determined with the Leave One Out procedure were the following:


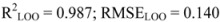


The detailed results of the LOO test are presented in Table 1.

**Table 1:** log *P_o/w_*, log *k’_0_* and related RP-HPLC log *P_o/w_* values for the reference compounds.

Name	Code	log *P_o/w_*^a^	log *k’_0_*	RP-HPLC log *k’*	LOO-predicted RP-HPLC log *P_o/w_*
2-Butanone	**1**	0.3	−0.06	0.21	0.15
Aniline	**2**	0.9	0.67	0.94	0.96
Acetanilide	**3**	1.0	0.88	1.15	1.19
Acetophenone	**4**	1.7	1.27	1.54	1.52
Benzene	**5**	2.1	1.87	2.14	2.14
Chlorobenzene	**6**	2.8	2.62	2.89	2.90
Bromobenzene	**7**	3.0	2.84	3.10	3.12
Naphthalene	**8**	3.6	3.15	3.42	3.36
Benzyl benzoate	**9**	4.0	3.74	4.01	4.01

^a^ Reference log *P_o/w_* values taken from OECD Guidelines.

Taking into account these values, it can be said that Equation (1) can be used to predict the log *P_o/w_* of QdO derivatives using the log *k’_0_* values.

#### 2.1.2. log *P_o/w_* of Quinoxalines di-*N*-Oxide

Retention times of QdO **10–29** were measured and the capacity factors (log *k’*) were calculated in varying proportions of methanol from 70% to 40%. The capacity factors were extrapolated to 0% methanol as in the case of the reference compounds (Table 2). The related log *P_o/w_* values were determined using Equation 6 and are also reported in Table 2.

**Table 2:** Capacity factors and related RP-HPLC log *P_o/w_* for QdO.

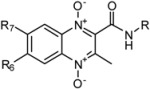			Capacity factors					RP-HPLC log *P_o/w_*
R	R_7_/R_6_	Comp.	log *k’_70_*	log *k’_60_*	log *k’_50_*	log *k’_40_*	log *k’_0_*	
C_6_H_5_-CH_2_	H/H	**10**	−0.64	−0.34	−0.05	0.28	1.49	1.76
	CH_3_/H	**11**	−0.56	−0.24	0.07	0.35	1.58	1.85
	Cl/H	**12**	−0.36	−0.05	0.30	0.65	1.98	2.25
	Cl/Cl	**13**	−0.11	0.27	0.66	0.98	2.46	2.72
2-phenylethyl	H/H	**14**	−0.58	−0.26	0.05	0.36	1.61	1.88
	CH_3_/H	**15**	−0.45	−0.13	0.21	0.47	1.73	2.00
	Cl/H	**16**	−0.37	0.00	0.36	0.69	2.11	2.38
	Cl/Cl	**17**	−0.06	0.34	0.74	1.07	2.60	2.87
*p*-BrC_6_H_4_-CH_2_	H/H	**18**	−0.24	0.11	0.48	0.85	2.30	2.57
	CH_3_/H	**19**	−0.16	0.21	0.60	0.94	2.43	2.69
	Cl/H	**20**	0.02	0.42	0.84	1.22	2.83	3.09
	Cl/Cl	**21**	0.19	0.65	1.15	1.60	3.51	3.78
*p*-CH_3_C_6_H_4_-CH_2_	H/H	**22**	−0.44	−0.10	0.25	0.62	2.02	2.29
	CH_3_/H	**23**	−0.36	−0.01	0.34	0.66	2.02	2.29
	Cl/H	**24**	−0.25	0.15	0.54	0.93	2.51	2.77
	Cl/Cl	**25**	0.14	0.54	0.93	1.33	2.91	3.18
2,2-diphenylethyl	H/H	**26**	−0.33	0.14	0.62	1.06	2.92	3.18
	CH_3_/H	**27**	−0.12	0.33	0.80	1.21	3.02	3.28
	Cl/H	**28**	−0.13	0.38	0.91	1.38	3.42	3.69
	Cl/Cl	**29**	0.25	0.76	1.31	1.78	3.86	4.12

Examination of the data indicates the influence of the quinoxaline structure on the log *P_o/w_* values. As expected, the log *P_o/w_* values of all the QdO derivatives are positive in a range between 1.5 and 4.5. The 20 compounds can be divided into five different series based on structure. Within each series of analogues, the insertion of a methyl group resulted in a soft increase of the log *P_o/w_* value, and replacing the hydrogen atom with one or two chloro groups supposed an increase of 0.5 or 1.0 unit, respectively. Increasing the aliphatic chain between the amide group and the aromatic system resulted in an increase of the log *P_o/w_* values as can be observed comparing derivatives **10**, **11**, **12**, **13** *versus* **14**, **15**, **16**, **17**. The *p*-bromo substituent (compounds **18–21**) increases the lipophilicity of the molecules. Finally, looking at the values of compounds **26**, **27**, **28** and **29**, it can be observed that these compounds, which contain a diphenyl substituent on the amide chain, presented the highest log *P_o/w_* values of all the QdO derivatives.

Therefore, the results suggest that the HPLC analysis is a suitable method for determining the log *P_o/w_* for quinoxaline derivatives instead of classical methods which are too slow, labor intensive and expensive. In particular, the RP-HPLC method for the QdO derivatives allows avoiding the problems associated with the classical shake-flask method, impossible to use with this kind of compounds.

### 2.2. Calculated log P_o/w_

All of the QdO were subjected to the ALOGPS online module. Seven values were obtained for each compound using different computational methods included in this module. The predicted log *P_o/w_* data are reported in Table 3. RMSE was calculated for each computational method in order to judge which method best suits experimental RP-HPLC.

The RMSE calculated from experimental and estimated log *P_o/w_* values ranged between 0.36 and 3.52 for XLOGP3 and MLOGP, respectively. From these data, XLOGP3 can be considered as the best approach to estimate log *P_o/w_* for the QdO.

**Table 3:** Calculated log *P_o/w_* for quinoxaline derivatives and related RMSE.

Comp.	RP-HPLC log *P_o/w_*	Calculated log *P_o/w_*						
		ALOGPs	miLOGP	ALOGP	MLOGP	LogK_OW_	XLOGP2	XLOGP3
**10**	1.76	0.43	−0.48	1.11	−1.71	0.55	5.03	1.28
**11**	1.85	0.50	−0.05	1.60	−1.47	1.10	5.46	1.64
**12**	2.25	1.03	0.18	1.77	−1.20	1.20	5.65	1.90
**13**	2.72	1.72	0.78	2.44	−0.70	1.84	6.27	2.53
**14**	1.88	0.63	−0.07	1.43	−1.47	1.04	5.18	1.74
**15**	2.00	0.80	0.35	1.92	−1.24	1.59	5.62	2.10
**16**	2.38	1.25	0.58	2.09	−0.97	1.69	5.80	2.37
**17**	2.87	2.03	1.19	2.76	−0.47	2.33	6.43	2.99
**18**	2.57	1.14	0.33	1.86	−1.08	1.44	5.82	1.97
**19**	2.69	1.29	0.76	2.34	−0.85	1.99	6.26	2.33
**20**	3.09	1.82	0.99	2.52	−0.59	2.09	6.44	2.60
**21**	3.78	2.74	1.59	3.19	−0.09	2.73	7.07	3.22
**22**	2.29	0.56	−0.03	1.60	−1.47	1.10	5.46	1.64
**23**	2.29	0.75	0.40	2.08	−1.24	1.65	5.90	2.01
**24**	2.77	1.22	0.63	2.26	−0.97	1.74	6.08	2.27
**25**	3.18	2.31	1.07	2.71	−0.36	2.16	6.71	2.90
**26**	3.18	1.78	1.31	2.78	−0.35	2.16	6.67	3.19
**27**	3.28	1.98	1.74	3.27	−0.14	2.71	7.11	3.56
**28**	3.69	2.36	1.97	3.45	0.12	2.81	7.29	3.82
**29**	4.12	3.19	2.57	4.11	0.60	3.45	7.92	4.45
**RMSE**		1.28	1.96	0.43	3.52	0.89	3.47	0.36

Nevertheless, and in an attempt to improve the predictive capacity of the ALOGPS, the LIBRARY mode was used, and experimental log *P_o/w_* data of compounds **10–13** were used to generate the library that was taken into consideration for recalculating the log *P_o/w_* of the rest of QdO (Table 4).

**Table 4:** Calculated log *P_o/w_* for quinoxaline derivatives using ALOGPS with and without LIBRARY mode.

Comp.	RP-HPLC log *P_o/w_*	ALOGPs	ALOGPs LIBRARY
**14**	1.88	0.63	1.93
**15**	2.38	1.25	2.43
**16**	2.00	0.80	2.08
**17**	2.87	2.03	3.04
**18**	2.57	1.14	2.40
**19**	3.09	1.82	2.92
**20**	2.69	1.29	2.54
**21**	3.78	2.74	3.54
**22**	2.29	0.56	1.90
**23**	2.77	1.22	2.41
**24**	2.29	0.75	2.06
**25**	3.18	2.31	3.19
**26**	3.18	1.78	3.18
**27**	3.69	2.36	3.67
**28**	3.28	1.98	3.33
**29**	4.12	3.19	4.32
**RMSE**		1.29	0.19

The examination of the data reveals that ALOGPS LIBRARY mode presents a RMSE of 0.19, making it the best approach for predicting the partition coefficients of QdOs.

#### *In Silico* Screening of the QSAR Relationship

The anti-tuberculosis activity of the studied compounds has been previously reported [40,41]. *In vitro* evaluation of the anti-tuberculosis activity has been carried out within the Tuberculosis Antimicrobial Acquisition & Coordinating Facility (TAACF) screening program for the discovery of novel drugs for the treatment of tuberculosis. The compounds were tested against *Mycobacterium tuberculosis* H37Rv (ATCC 27294) in BACTEC 12B medium using the Microplate Alamar Blue Assay (MABA). Compounds showing an IC_90_ value of ≤10 µg/mL were considered “Active” for antitubercular activity and considered for the VERO cell cytotoxicity assay. Cytotoxicity is determined as the CC_50_ using a curve fitting program. Ultimately, the CC_50_ is divided by the IC_90_ to calculate a SI (Selectivity Index) value. SI values of ≥10 are considered “Active”.

Since log *P_o/w_* is an important ADME parameter, a correlation with the anti-tuberculosis activity of compounds was looked for. However, it was verified that there is not a strong relationship (R^2^ < 0.2) between log *P_o/w_* and activity, expressed as log(1/IC_50_) or log(1/IC_90_). In spite of this, it seems that a low value of log *P_o/w_* could be related with better values of anti-tuberculosis activity.

This fact could be explained as a consequence of the structure of the *M. tuberculosis* envelope structure. Porin presented in the membrane control the diffusion of small hydrophilic molecules; and, therefore, *M. tuberculosis* is more permeable to hydrophilic drugs such as INH, PZA, EMB or gatifloxacin that present a log *P* of −0.71, −0.71, −0.12 and −0.23, respectively (ALOGPS, [42]) In this sense, lipophilic molecules should be able to easily cross the lipid bilayer; however, the bilayer’s uncommon thickness and the presence of the mycolic acids seem to dicrease the permeability to lipophilic drugs. Nevertheless, it has been observed that the more lipophilic the agents are, the more active they usually are against *M. tuberculosis*, as for instance, PAS, RIF or rifapentine presenting log *P* values of 0.62, 3.85 and 4.83, respectively (ALOGPS). This fact suggests that there must be an specific pathway for lipophilic drug transport [43].

Recent studies have focused on the influence of physicochemical properties of antibacterial drugs and they have concluded that it is not possible to establish a strong relationship between these properties and the anti-tuberculosis activity. In this sense, it seems that much work is needed in order to understand *M. tuberculosis* and its metabolism. At this moment, recent reviews affirm that antibacterial drugs constitute an special physicochemical space completely different from the space covered by drugs in many other therapeutic areas [44].

On the other hand, in the previous paper a good relationship was found between redox peak potential E_pc,1_ and activity [45]. In that case, however, the correlation was found within an almost homogeneous series of molecules. Here a more heterogeneous set of compounds has been studied. For molecules **11–24**, **26–29** there is not a strong relationship (R^2^ < 0.2) between peak potential and activity. However, it is evident that the compounds roughly align (according to the R substituent) or group themselves (according to the R_6_ and R_7_ substituents) in homologous series. This behaviour justifies the previously found relationship for a small and almost uniform set of molecules.

The above said results point out that log *P_o/w_* and E_pc,1_, although important parameters, cannot explain alone the observed activity. When considering simultaneously the effect of both factors, a two-variable model was built to predict activity, resulting in a better fitting ability (R^2^ ≅ 0.53). Some other variable is probably needed to improve the model. 

In order to enlarge the set of possible variables, the structures of compounds **10–29** were optimised with the MOE (Molecular Operating Environment) software using a MMFF force field [46]. From the optimised structures 333 molecular descriptors were calculated. This set of parameters was reduced to 269 excluding variables with constant values. A multiple linear regression (MLR) procedure with a stepwise forward selection of the variables was applied to the whole set of variables (269 from MOE plus the experimental log *P_o/w_* and redox peak potential). This resulted in a two-variable model (R^2^ ≅ 0.8) both for log(1/IC_50_) and log(1/IC_90_).

One selected descriptor (vsurf_HB6) belongs to the “Surface Area, Volume and Shape Descriptors” family and is related to the *H*-bond donor capacity. The other descriptor (SlogP_VSA4) belongs to the “Subdivided Surface Areas” group and is related to an approximate accessible van der Waals surface area calculation for each atom, along with the contribution to log *P_o/w_* (as calculated in MOE) for each atom (see Supporting Information).

The addition of both experimental log *P_o/w_* and E_pc,1_ to the previously selected two descriptors allowed us to build a four-variable model with R^2^ = 0.85 for log(1/IC_50_) and R^2^ = 0.84 for log(1/IC_90_).

In conclusion, the reported statistical analysis confirms the importance of the reduction peak potential E_pc,1_ in the definition of the cytotoxic activity. This reduction process has been demonstrated to be consistent with reduction of the *N*-oxide functionality to form a reactive radical anion, which could lead further to superoxide ion or other toxic oxy radical species responsible for the biological activity [45]. However, in order to obtain a more general QSAR relationship, three other descriptor should be considered. Although the relatively small number of compounds considered, with respect to the large set of descriptors, limited the fitting ability of the model (R^2^ ≥ 0.84), the lipophilicity, the *H*-bond donor capacity and a descriptor related to the molecular dimensions were found to be involved in the modulation of the final biological activity. In particular, on the basis of the regression coefficient, the redox properties (E_pc,1_) correlate positively with both log(1/IC_50_) and log(1/IC_90_); this means that an easy reduction increases the activity. On the contrary, the lipophilicity and shape descriptors affect the activity negatively.

## 3. Experimental Section

### 3.1. Chemical Synthesis

**Scheme 1:**
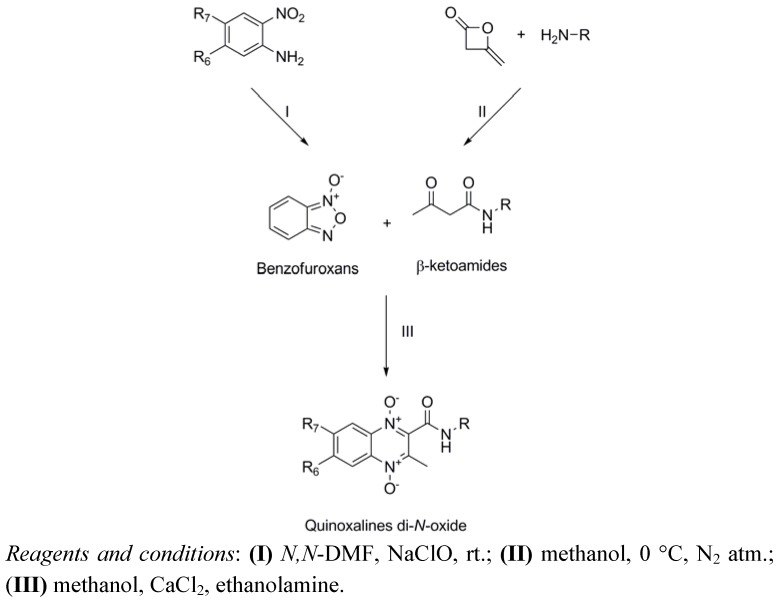
General synthetic scheme.

The synthesis of the quinoxaline 1,4-di-*N*-oxide derivatives was carried out by a variation of the Beirut reaction [36,47,48,49,50,51], where the appropriate benzofuroxan (BFX) reacts with the corresponding β-ketoamide in the presence of calcium chloride and ethanolamine as catalysts (Scheme 1). The methods for the synthesis of quinoxaline-2-carboxamide 1,4-di-*N*-oxide derivatives and the structure characterization were reported elsewhere [40,41].

### 3.2. RP-HPLC Method

The RP-HPLC methods of Minick [52] and Lombardo [53] were considered for estimating the *n*-octanol/water partition coefficients (log *P*_o/w_) of QdO. The inorganic salts and the reference compounds were, at least, of analytical grade (Sigma Aldrich and Fluka) and used without further purification. HPLC-grade methanol, *n*-octanol (Sigma-Aldrich) and bi-distilled water were used to prepare the mobile phase. The retention times (t_R_) were measured using a Waters 2695 Separation Module system and a Waters 2487 Dual λ Absorbance Detector with Empower Pro Software.

The Supelcosil LC-ABZ column (5 μm, 15 cm × 4.6 mm) [18,52,53,54] was selected to substitute the organic phase of the shake-flask method because it has been reported that this column affords a reasonable correlation model for a great variety of compounds [18,22,49,55]. The mobile phase consisted of 20 mM 3-morpholinopropanesulfonic acid (MOPS) buffer (pH 7.4) and methanol in varying proportions, from 70 to 40%. *n*-Octanol (0.25%) was added to methanol, and *n*-octanol-saturated water was used to prepare the buffer [41]. The other chromatographic conditions were: flow 1.0 mL/min^−1^; isocratic elution, UV-visible detector set at the wavelength with maximum absorbance (260 and 210 nm). The test solutions were 0.3 mM in the desired compound. All the experiments were performed at 25 ± 2 °C at least twice.

The t_R_ of each QdO derivative **10–29** was measured at different proportions of methanol (from 70 to 40%) and injections of pure methanol were used to determine the column dead-time (t_0_). The capacity factors were calculated according to Equation (2):


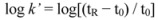
(2)

Starting from these results, the extrapolation to 0% methanol for each compound was calculated and the capacity factors were used to predict the corresponding log *P_o/w_*.

The capacity factors of compounds **1–9**, with known log *P_o/w_* [16,20,39] and accepted as reference compounds by the OECD [39], were used to create a calibration curve [Equation (3)]:


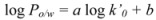
(3)

The common coefficient of determination R^2^ was used to evaluate the fitting ability of the model. Another measurement for defining the accuracy of the proposed model is the RMSE (Root Mean Squared Error), which summarizes the overall error of the model [Equation (4)]:

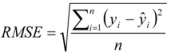
(4)
where *ŷ_i_* is the log *P_o/w_* value calculated by Equation (2), *y_i_* is the reference value and *n* is the number of reference compounds used to create the curve.

### 3.3. Cross-Validation of the RP-HPLC Method

In order to judge if the experimentally measured log *k’_0_* can be used to predict the log *P_o/w_* value, a calibration curve was created with the capacity factors of the reference compounds and a linear regression equation between the log *P_o/w_* and log *k’_0_* was determined.

The robustness of the model and its predictivity were evaluated by the leave-one-out (LOO) cross-validation procedure [28,56]. According to this procedure, the log *P_o/w_* value of each compound in the reference data set is predicted by the equations derived from all the other compounds except the predicted one. At the end of this procedure two values are available for each reference compound, the reference shake-flask log *P_o/w_* value (*y_i_*) and the predicted one (*ŷ_i_*). With these data, two statistical parameters (R^2^_LOO_, RMSE_LOO_) were calculated to indicate the predictivity of the model. The regression coefficient (R^2^_LOO_) is defined by Equation (5):

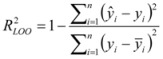
(5)
where 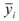 is the mean of the reference shake-flask log *P_o/w_* value and *n* is the number of reference compounds. A high value of R^2^_LOO_ indicates a good predictive ability of the model. The root mean squared error in prediction (RMSE_LOO_) is calculated with Equation (6):

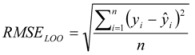
(6)


### 3.4. log P_o/w_ Predictive Approaches

All of the synthesized QdO were subjected to the ALOGPS module with the aim of comparing experimental log *P_o/w_* values and predicted data. The ALOGPS method is part of the ALOGPS 2.1 program and provides interactive on-line predictions of log *P_o/w_* and aqueous solubility. log *P_o/w_* values can be obtained from different computation methods: ALOGPS 2.1 includes, among other calculation programs, ALOGPs, miLogP, ALOGP, MLOGP, KOWWIN, XLOGP2 and XLOGP3 [42].

The lipophilicity calculations within ALOGPS 2.1 are based on the associative neural network approach and an efficient partition algorithm. ALOGPs was developed with 12,908 molecules from the PHYSPROP database using 75 E-state indices. 64 neural networks were trained using 50% of molecules selected by chance from the whole set. The log *P_o/w_* prediction accuracy is RMSE = 0.35 and standard mean error s = 0.26 [33].

This program also provides a possibility to include new data into the memory of neural nets without retraining the neural networks themselves in the so-called LIBRARY mode. The LIBRARY dramatically improves prediction of the ALOGPS program for the log *P_o/w_* prediction using in-house data sets [21,33,35]. The LogKow (Kow-WIN) program estimates the log *P_o/w_* of organic compounds and drugs using an atom/fragment contribution method developed at Syracuse Research Corporation [57]. The miLogP is calculated by the methodology developed by Molinspiration as a sum of fragment-based contributions and correction factors. This method for log *P_o/w_* prediction is based on group contributions which have been obtained by fitting calculated log *P_o/w_* with experimental log *P_o/w_* for a training set of more than twelve thousand, mostly drug-like molecules. Molinspiration methodology [58] for log *P_o/w_* calculation is very robust and capable of processing practically all organic and most organometallic molecules XLOGP2 gives log *P_o/w_* values by summing the contributions of component atoms and correction factors. Altogether 90 atom types are used to classify carbon, nitrogen, oxygen, sulfur, phosphorus and halogen atoms, and 10 correction factors are used for some special substructures. The contributions of each atom type and correction factor are derived by multivariate regression analysis of 1853 organic compounds with known experimental log *P_o/w_* values [34]. The additive model implemented in XLOGP3 uses a total of 87 atom/group types and two correction factors as descriptors. It is calibrated on a training set of 8,199 organic compounds with reliable log *P_o/w_* data through a multivariate linear regression analysis [26]. ALOGP, also known as “Ghose-Crippen octanol-water partition coefficient”, is a log *P_o/w_* calculated with the Ghose-Crippen contribution method based on hydrophobic atomic constants measuring the lipophilic contributions of atoms in the molecule, each described by its neighbouring atoms [59]. MLOGP, also known as “Moriguchi octanol-water partition coefficient”, expresses log *P_o/w_* in terms of 13 structural parameters.

## 4. Conclusions

In this study the RP-HPLC retention times of 20 QdO derivatives were measured and it was found that highly accurate log *P_o/w_* values can be predicted from the experimental log *k’_0_* by using the linear regression equation relating log *P_o/w_* and log *k’_0_* (Equation 1). Consequently, the results suggest that the determination of log *P_o/w_* for QdO can be successfully carried out by measuring the capacity factors based on the simple RP-HPLC method. This method allows us to avoid the experimental problems presented by the classical shake-flask method when trying to measure the partition coefficients of QdO derivatives that present low stability in aqueous solution and create emulsions during partitioning.

After a comparison among different methods for the calculation of log *P_o/w_*, in cases where no experimental data is available, XLOGP3 is proposed as the best program to calculate the log *P_o/w_* values for the QdO presented in this work (RMSE = 0.36). On the other hand, the ALOGPS LIBRARY method, implemented with the experimental data, predicts log *P_o/w_* values that match the experimental ones with the lowest RMSE (0.19).

Finally, a preliminary statistical analysis confirms the importance of the reduction peak potential E_pc,1_ in the definition of the cytotoxic activity of QdO. Moreover, the lipophilicity, the *H*-bond donor capacity and a descriptor related to the molecular dimension were found to also be involved in the modulation of the final biological activity.

## Supplementary Materials

Supplementary File 1
