# Supplementary material for: Studies on Log Po/w of Quinoxaline di-N-Oxides: A Comparison of RP-HPLC Experimental and Predictive Approaches
Source: Molecules. 2011 Sep 13;16(9):7893–908. doi: 10.3390/molecules16097893 (PMC6264295; doi:10.3390/molecules16097893)
Supplement: Supplementary File 1 [file molecules-16-07893-s001.pdf]

Supplementary Material

Studies on log $P_{o/w}$ of Quinoxalines di-*N*-Oxide: A Comparison of RP-HPLC Experimental and Predictive Approaches

Elsa Moreno [1,2,*], Elisabetta Gabano [2], Enrique Torres [1], James A. Platts [3], Mauro Ravera [2], Ignacio Aldana [1], Antonio Monge [1] and Silvia Pérez-Silanes [1]

[1] Neglected Diseases Section, Drug R&D Unit, Center for Applied Pharmacobiology Research, University of Navarra, C/ Irunlarrea s/n, 31008 Pamplona, Spain

[2] Dipartimento di Scienze dell'Ambiente e della Vita, Università del Piemonte Orientale "A. Avogadro", Viale Michel 11, 15121 Alessandria, Italy

[3] School of Chemistry, Cardiff University, Park Place, Cardiff CF10 3AT, UK

* Author to whom correspondence should be addressed; E-Mail: emoreno4@alumni.unav.es; Tel.: +34-948-425653; Fax: +34-948-425652.

Stability of Quinoxalines di-*N*-Oxide in Aqueous Solution

Photochemical instability of QdO is well-known and many studies have been reported. It has been observed that the absorption spectrum of quinoxalines di-*N*-oxide neutral solutions change quickly due to exposure to sunlight. Previous reports suggest that the photolysis of these derivatives consists in the interconversion of quinoxaline 1,4-dioxide to 2*H*-quinoxaline 4-oxide through an oxaziridine intermediate (Scheme A1). Furthermore, it has been concluded that oxaziridine intermediate also reverts back to quinoxaline 1,4-dioxide and the equilibrium is forced to 2-oxo-1,2-di*H*-quinoxaline 4-oxide in acidic solution and alcoholic solutions. For these reasons, QdO solutions must be kept protected from light and used as soon as prepared.

Scheme A1. Photolysis of QdO.

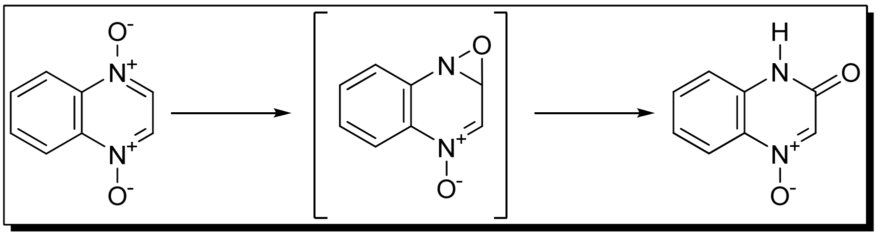

With the aim of studying the availability of the shake-flask method, four compounds (**10**, **14**, **22** and **26**) were selected and many attempts to measure the log $P_{o/w}$ were performed using the classical shake-flask method. As expected, the UV spectra of the QdO derivatives changed during the experiments, and for this reason, the observed UV data could not be considered to determine the log $P_{o/w}$ values.

With the aim of studying the degradation of QdO derivatives, compound **22** was selected and its HPLC chromatograms were studied. As can be observed in Figure A1, quinoxalines present two different wavelengths with maximum absorbance. It can be said that the absolute maximum is within

the range of 250–260 nm; and two maxima can be often observed in this area (220–230 and 250–260 nm). Another characteristic maximum appears close to 360 nm.

Figure A1. UV spectrum of compound **22**.

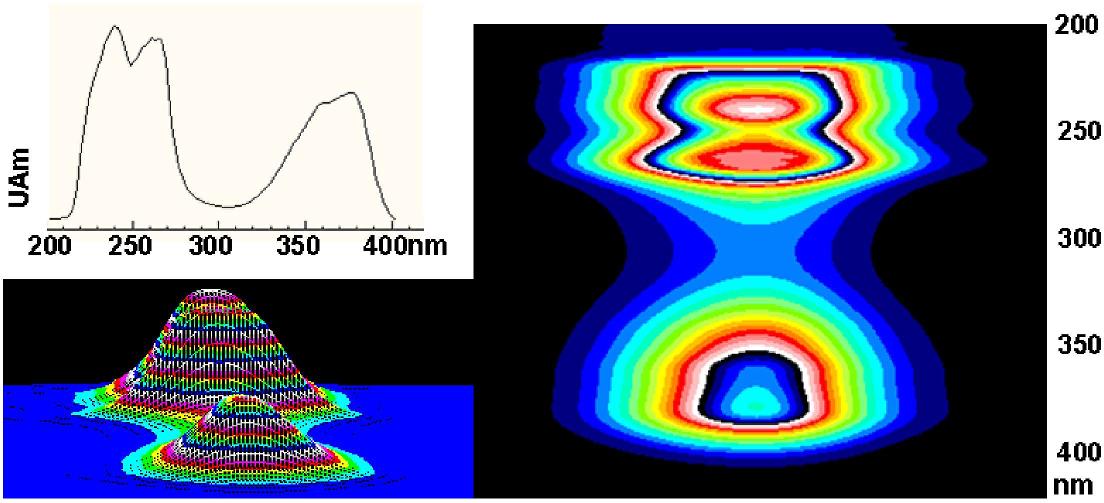

Once the UV spectrum of QdO derivatives was studied, the UV spectra of compound **22** were studied, over a pH range from 1.0 to 11.0. As can be observed in Figure A2, fresh aqueous solution of compound **22** presents the characteristic spectrum previously explained.

Figure A2. UV spectrum of fresh aqueous solution of compound **22**.

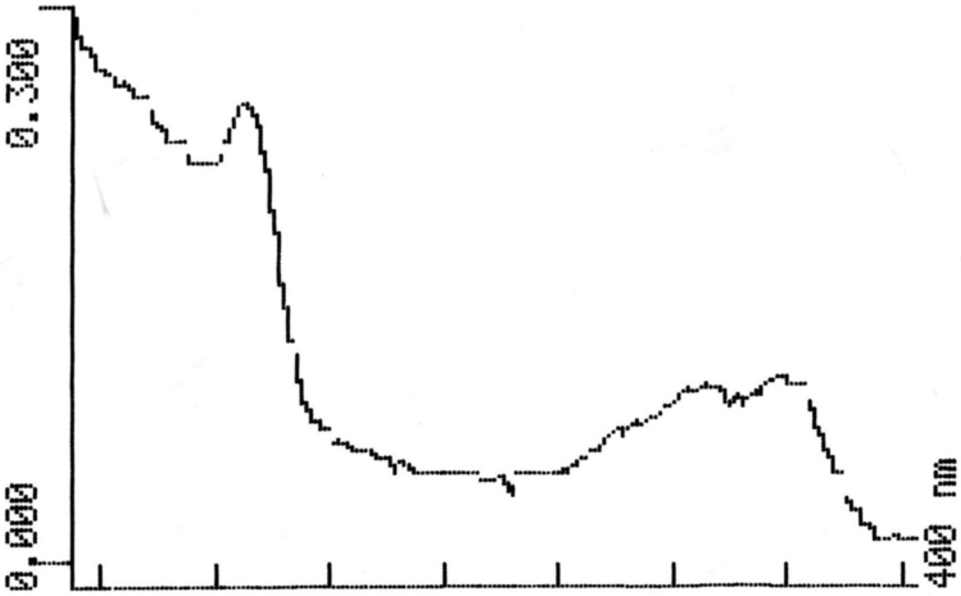

After 48 h, the UV spectra show that the quinoxaline has been degraded as the maxima that appeared at nearly 220 and 260 nm have disappeared. Moreover, the more extreme the pH is, the more the UV spectrum changes (Figure A3).

Figure A3. UV spectrum of compound **22** (pH ranging from 1 to 11).

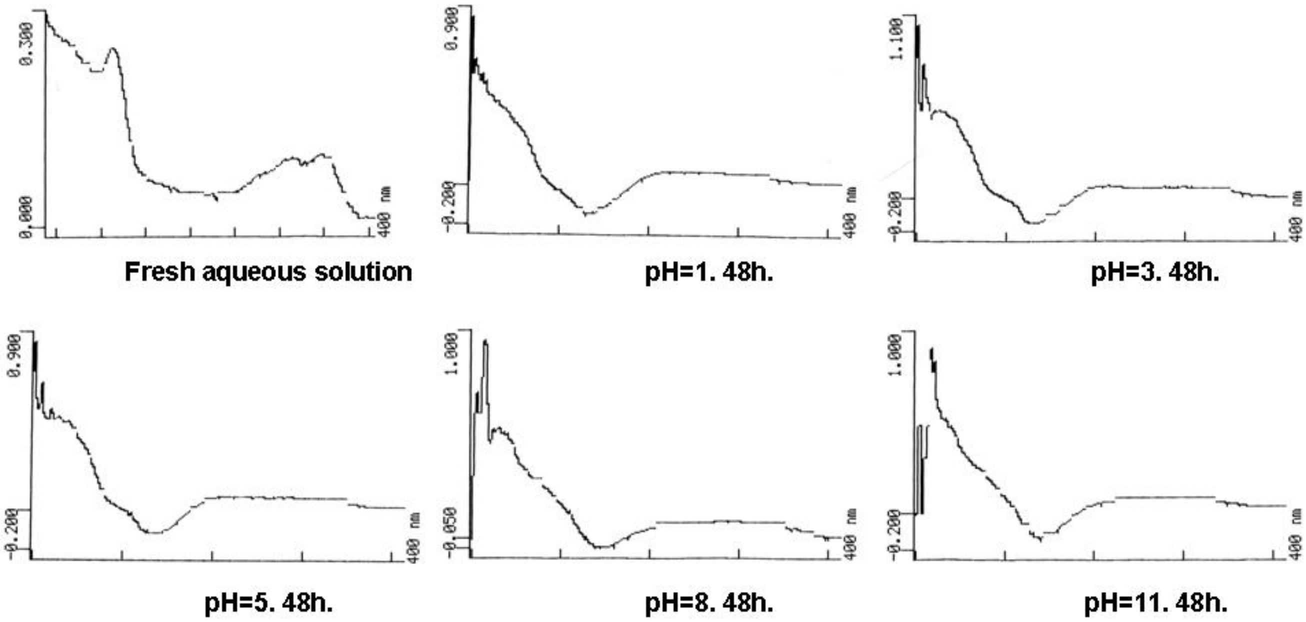

In conclusion, no reliable results can be obtained with the shake-flask method. Therefore, this method is not suitable for quinoxaline di-*N*-oxide derivatives. Nevertheless, this fact does not affect the experimental RP-HPLC method because this method is performed at pH = 7.4 and quinoxalines di-*N*-oxide derivatives are more stable in neutral solutions than at extreme pH. Moreover, the period of time in which the compounds are in contact with the mobile phase is not long enough to degrade the quinoxalines, as observed when studying the corresponding chromatograms.

Chromatograms were developed on an Ultimate 3,000 Chromatograph (DIONEX) with Chromeleon v.6.8 software. The measurements were performed using an RP 18 column (LICHROSPHER 100 RP 18 E.C. 5 µm; 10 × 0.46; TEKNOKROMA) as the stationary phase, at a flow rate of 1 mL/min and with methanol/water (60:40) as the mobile phase. Compound **22** was dissolved in methanol. UV spectra swere studied on a BIO RAD SMARSPEC™ PLUS spectrophotometer at wavelengths from 400 to 200 nm.

QSAR Analysis

Table A1. Values of experimental $\log(1/IC_{50})$ and $\log(1/IC_{90})$ and values of the selected molecular descriptors for the complete data set of complexes **10–29**.

Compound	$\log(1/IC_{50})$	$\log(1/IC_{90})$	$E_{pc,1}$	SlogP_VSA4	Vsurf_HB6
**10**	−0.182	−0.417	–	32.561	17.875
**11**	−0.431	−0.624	−1.658	35.747	18.250
**12**	0.420	0.367	−1.482	32.561	17.875
**13**	0.699	0.699	−1.211	32.561	17.125
**14**	−0.250	−0.484	−1.625	32.561	16.625
**15**	−0.657	−0.936	−1.649	35.747	16.750

Table A1. *Cont.*

Compound	log(1/IC50)	log(1/IC90)	Epc,1	SlogP_VSA4	Vsurf_HB6
**16**	0.699	0.699	−1.525	32.561	16.625
**17**	0.721	0.721	−1.387	32.561	17.250
**18**	−1.069	−1.193	−1.540	32.561	19.625
**19**	−1.532	−1.893	−1.580	35.747	20.625
**20**	−0.597	−0.727	−1.445	32.561	19.250
**21**	−0.782	−0.840	−1.363	32.561	20.375
**22**	−0.792	−0.830	−1.580	35.747	18.500
**23**	−1.912	−2.000	−1.590	38.932	18.500
**24**	−1.316	−1.506	−1.489	35.747	17.625
**25**	−2.000	−2.000	−	35.747	17.750
**26**	−1.064	−1.204	−1.591	35.747	18.500
**27**	−1.150	−1.225	−1.640	38.932	14.750
**28**	−1.385	−1.781	−1.491	35.747	19.375
**29**	−1.744	−1.823	−1.410	35.747	20.875

Final QSAR Models

- $\log(1/IC_{50}) = 16.05 - 0.31\ \log P_{o/w} + 2.15\ E_{pc,1} - 0.27\ \text{vsurf\_HB6} - 0.22\ \text{SlogP\_VSA4}$
  $R^2 = 0.85$; RMSE = 0.37
- $\log(1/IC_{90}) = 17.29 - 0.32\ \log P_{o/w} + 2.73\ E_{pc,1} - 0.29\ \text{vsurf\_HB6} - 0.22\ \text{SlogP\_VSA4}$
  $R^2 = 0.84$; RMSE = 0.41
